# Chlordetect: Commercial Calcium Aluminate Based Conductimetric Sensor for Chloride Presence Detection

**DOI:** 10.3390/s17092099

**Published:** 2017-09-13

**Authors:** Magda Torres-Luque, Johann F. Osma, Mauricio Sánchez-Silva, Emilio Bastidas-Arteaga, Franck Schoefs

**Affiliations:** 1 CAPACITÉS, SAS. 26 Boulevard Vincent Gâche, 44200 Nantes, France; 2 CMUA, Department of Electrical and Electronics Engineering, Universidad de los Andes, Carrera 1E N, 19A-40 Edificio ML, Piso 7. 111711 Bogotá, Colombia; jf.osma43@uniandes.edu.co; 3 Department of Civil and Environmental Engineering, Universidad de los Andes, Carrera 1E N, 19A-40 Edificio ML, Piso 6. 111711 Bogotá, Colombia; msanchez@uniandes.edu.co; 4 Institute for Research in Civil and Mechanical Engineering (GeM, CNRS UMR 6183), Sea and Littoral Research Institute (IUML, CNRS FR 3473), Université de Nantes, Centrale Nantes, 2 rue de la Houssinière BP 92208, 44322 Nantes, France; Emilio.Bastidas@univ-nantes.fr (E.B.-A.); franck.schoefs@univ-nantes.fr (F.S.)

**Keywords:** chloride concentration, chloride sensors, monocalcium aluminate, dielectric spectroscopy, corrosion, dielectric materials

## Abstract

Chloride presence affects different environments (soil, water, concrete) decreasing their qualities. In order to assess chloride concentration this paper proposes a novel sensor for detecting and measuring it. This sensor is based on electric changes of commercial monocalcium aluminate (CA) when it interacts with chloride aqueous solutions. CA is used as a dielectric material between two coplanar capacitors. The geometry proposed for this sensor allows to assess the chloride content profile, or to make four times the same measurement. Besides, the experimental design gives us the possibility of study not just the chloride effect, but also the time and some geometric effects due to the sensor design. As a result, this sensor shows a limit of detection, sensitivity, and response time: 0.01 wt % Cl^−^ and 0.06 wt % Cl^−^, and 2 min, respectively, comparable with other non invasive techniques as optical fibre sensors.

## 1. Introduction

In civil engineering, agriculture, domestic, and in industrial fields measuring chloride concentration is a necessity. Chloride ingress is one of the major causes of reinforced concrete (RC) deterioration affecting structural serviceability and safety. Chloride assessing can provide useful information for predicting, assessing, maintaining, and controlling lifetime, reliability, health and durability of RC structures [1]. In agriculture, measuring soil salinity is important to determine the land productivity and its economic impact, especially in those regions where rising water has caused changes on soil surface due to salt content [2]. Sakai et al. [3] have shown the importance of water quality assessment through multichannel sensors. Taking into account multiple pollutant agents (pesticides, industry wastes, etc.) to evaluate the quality of drinking water is necessary in order to prevent health–risks [4].

Until now, some chloride sensors have been developed based on different physical principles (e.g., optical fiber, electrical resistance and ion selective electrode). The most known are those that are based on ion selective electrode technology (ISE). They can be used in diverse fields under hard conditions (soil and water quality). However, for specific conditions, such as those in RC structures (e.g., high alkalinity, high humidity, corrosive ions, residual stresses, etc.), these sensors must be modified to make them more robust and stable [5,6]. On the other hand, optical fiber and resistivity sensors have shown good potential. They withstand hard environments and provide more information than ISE sensors regardless the geometry. However, their costs are high, and their measurements shift when temperature or/and humidity change [1]. In fact, resistivity sensors are sensitive to chemical factors, e.g., the presence of other ions than chloride (CO_2_); therefore, the data acquisition system to identify the different signals is expensive [7].

According to previous work [1], where the most important developed sensors were evaluated, there are five challenges in sensor developing: (i) Independence of environmental actions; (ii) independence of geometry and interferences; (iii) multimeasurement ability; (iv) chemical stability, durability and maintainability; and (v) costs. In the way to overcome these challenges, this paper present a novel sensor based on the electric capacitance variation of monocalcium aluminate (CA): The chlordetect (patent [8]). Due to the materials used and fabrication process, this sensor is not only resistant to aggressive environments, but also it is an inexpensive sensor.

This paper is structured as follows. Section 2 presents basic theoretical and practical knowledges about capacitance and impedance analysis, and also chemical bases of commercial monocalcium aluminate. Section 3 shows the suitability of the CA for detecting chloride presence in the environment through the experimental design and some important results of the characterisation. Section 4 describes the sensor design, its suitability and its performance facing chloride aqueous solutions. Finally, Section 5 presents the principal findings of this study and some challenges for the future.

## 2. Theoretical Aspects of the Sensor

### 2.1. Conductimetric Sensors

**Figure 1:**
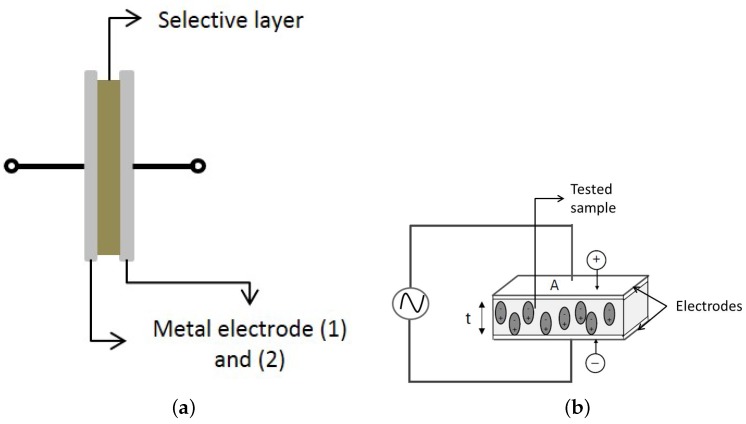
Scheme of (**a**) Conductimetric sensor and (**b**) Dielectric spectroscopy technique.

Because of their low costs of fabrication, conductimetric sensors are used for many tasks, for example for measuring suspended sediment concentration in soil and water [9], or for sensing small organic molecules [10]. Conductimetric sensors are usually bipolar devices (Figure 1a) in which the conductance (inverse of the impedance) of the cell is measured at both terminals, and it assesses changes in the selective layer.

Impedance ($Z_{\left(\omega \right)}$) has two parts: Reactance ($X_{\left(\omega \right)}$) and resistance (*R*, and it does not depend on frequency). While the first one regards the capacitance behavior, the second one represents the electrical resistivity. Equation (1) shows the general equation of impedance.

(1) $$\begin{array}{ccc} Z_{\left(\omega \right)} & = & R-jX_{\left(\omega \right)}\\ X_{\left(\omega \right)} & = & \frac{1}{j\omega C}\\ R & = & \frac{V}{I} \end{array}$$

where $C_{\left(\omega \right)}$ is the capacitance, *V* is the voltage, and *I* is the electrical current.

**Figure 2:**
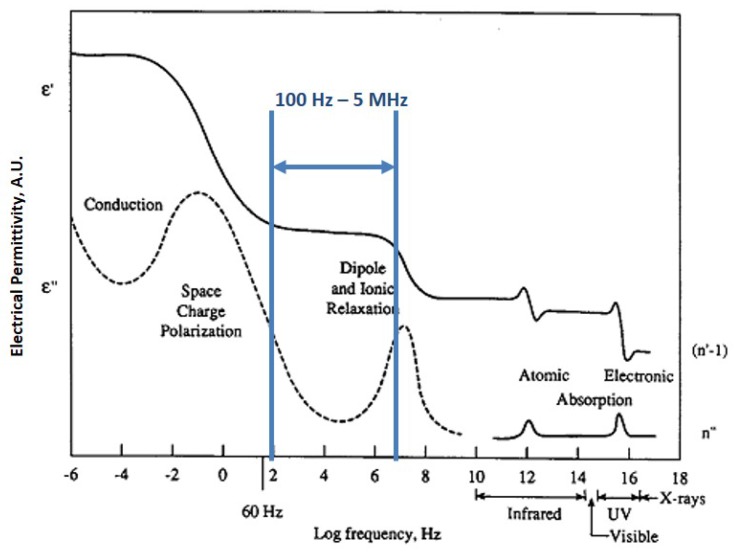
Dielectric response according to frequency. Adapted from [12].

In the first phase of this document the studied response is directly the capacitance that is affected by frequency (Figure 2), and in the second phase, the studied response is the impedance.

To choose the selective layer of the sensor, it is necessary to characterize different materials that can be sensitive to the chemical species of interest. This characterization can be done using dielectric spectroscopy (DS) that consists of measuring the electrical response (resistance and reactance) of a material when an external electric field is applied, Figure 1b. If the studied material has the ability to store energy, the material is classified as dielectric, it means that its dielectric constant is higher than that of the air.

Furthermore, depending on the arrangement of electric charge carriers that can be displaced (e.g., Cl^−^), every material has a different response as it is shown in Figure 2. In a microscopic level, several dielectric mechanisms can contribute to dielectric behavior; for example, ionic conduction strongly interact at microwave frequencies [11].

### 2.2. Commercial Monocalcium Aluminate (CA)

Calcium aluminates (CA, C_3_A, C_4_AF, C_12_A_7_, cement chemistry notation) are usually used in the steel industry as refractory mixed oxides, and in the cement community as hydraulic materials. Nowadays, other applications are under development such as optical devices, oxygen ionic conductors, and catalysts [13]. Aluminates such as CaO·Al_2_O_3_ (CA) and 3CaO·Al_2_O_3_ (C_3_A) are essential in cementitious materials because they are refractory binders [14]. Calcium Aluminate Cement (CAC) was patented by Lafarge in 1908. At the beginning it was used for RC structures; however, after rapid failure (cracking) of these structures, the use of CAC was restricted [15]. Nowadays, it is allowed in specific structures (e.g., sewer infrastructure, refractory concretes) or environments (e.g., low temperatures, acid environments) that require rapid settling or high strength at early stages [16].

According to [15,17,18,19] aluminate content in cements (Ordinary Portland Cement (OPC) and also CAC) is crucial in the chloride binding and diffusion. This can be explained because chlorides react with calcium aluminates in presence of water to form Friedel’s salts (Ca_4_Al_2_O_6_Cl_2_·10H_2_O) and other compounds. In general, chloride salts (e.g., CaCl_2_, NaCl or KCl) dissociate in water, and then react with aluminates. Chloride ions go inside hydrated aluminate structure ${\mathrm{Ca}}_{2}{\mathrm{Al}(\mathrm{OH})}_{6}^{+}$ replacing OH^−^ ions, and forming a distorted brucite-like main layers separated by interlayers of Cl^−^ and H_2_O molecules [13,20,21,22]. Normal process of Friedel’s salts formation is stated as follows [21]:

(2) $$\begin{array}{ccc} {\mathrm{Ca}(\mathrm{OH})}_{2}+2\mathrm{NaCl} & ⇌ & {\mathrm{CaCl}}_{2}+2{\mathrm{Na}}^{+}+2{\mathrm{OH}}^{-}\\ 3\mathrm{CaO}\cdot {\mathrm{Al}}_{2}O_{3}+{\mathrm{CaCl}}_{2}+{10H}_{2}O & ⟶ & {\mathrm{Ca}}_{4}{\mathrm{Al}}_{2}O_{6}{\mathrm{Cl}}_{2}\cdot {10H}_{2}O \end{array}$$

Although these reactions have been widely studied, their electrical changes still require further evaluation and analysis. One of these electrical changes is the electric polarization that affects dielectric constant and conductivity (resistivity). Polarization is the alignment of the dipoles in presence of an electric field, and causes stored charge increasing in the material.

Until now, the study of electrical parameters changes on construction materials (e.g., cement, concrete, and raw materials) through radio frequencies (3 Hz–300 GHz) have been used for detecting chloride [23], and dielectric parameters’ variation have been studied for determining changes on the microstructure of cements due to hardening and admixtures presence [24,25]. These experimental works use DS, a technique that applying an alternate voltage and measuring phase and magnitude of the current can determine the dielectric behavior variation [24]. Also, this technique has shown good potential for measuring chlorides in other materials, such as pork meat [26].

## 3. Phase I—Commercial Monocalcium Aluminate (CA) Characterization

Commercial monocalcium aluminate was provided by Parexlanko-France (2012), its commercial name is Ternal RG. To control and to understand the material’s properties and behavior, it has been characterized structurally and electrically. Following subsection explains the parameters for both characterization procedures.

### 3.1. Electrical Characterization

An impedance analyzer Agilent 4294A (Agilent, Santa Clara, CA, USA) and the dielectric text fixture 16451B were used to characterize the aluminates before, during and after they reacted with chlorides. The frequency range was between 100 Hz and 5 MHz (Figure 2) and the area (A) of the plates was $1.13\times 10^{-3}$ m^2^. This device works between 0 and 55 °C, and an open/short compensation was necessary.

According to Figure 2 this range is capable of showing the response of ionic and dipole relaxation due to applied electric field. As a result measuring capacitance in this range of frequencies gives us information about the presence of chloride ion and also water.

The CA powder were put in plastic containers where 6.6 g of the CA is laid and tamped with a rammer during 120 s until it reaches a thickness between $1.84\times 10^{-3}$ m and $2.27\times 10^{-3}$ m.

Water deionized (0 M) and three NaCl solutions were used to test the dielectrical behavior of CA: 0.5 M, 0.7 M and 1.0 M. Table 1 lists the name and characteristics of each test. In addition, measurements were performed 1 min after 1 mL of NaCl solution is added to CA and every 10 min for 1 h to study time-dependency. Each experiment was performed by triplicate.

**Figure 3:**
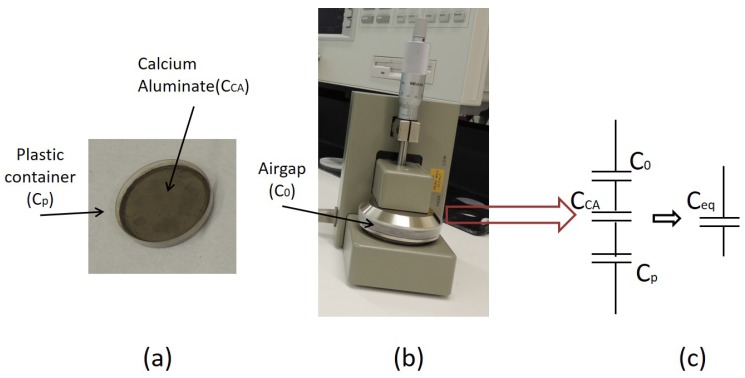
(**a**) CA sample in the container; (**b**) Sample located in the Dielectric text fixture 16451B; (**c**) Equivalent circuit ($C_{e}$).

The non-contacting electrode method was used [11]. The equivalent capacitor ($C_{eq}$) for this method, and real images are shown in Figure 3. The following equation was used to obtain the relative permittivity of the CA ($\varepsilon_{rCA}$) and CA with Cl solutions:

(3) $$\begin{array}{ccc} \frac{1}{C_{eq}} & = & \frac{1}{C_{p}}+\frac{1}{C_{CA}}+\frac{1}{C_{0}}\\ \varepsilon_{rCA} & = & \frac{C_{eq}C_{p}C_{0}}{C_{p}C_{0}-C_{0}C_{eq}-C_{p}C_{eq}}*\frac{t_{CA}}{\varepsilon_{0}A} \end{array}$$

where $C_{p}$ is the capacitance of the plastic container, $C_{0}$ is the capacitance of the airgap, $t_{CA}$ is the thickness of the material and $\varepsilon_{0}$ is the vacuum permittivity. For this experiment $C_{0}$ is estimated by measuring, with the same instrument, the capacitance of the airgap used during experiments that was 0.4 mm. Likewise, $C_{p}$ was determined by measuring the capacitance of the empty plastic container.

In summary, Table 2 shows the variables that were considered in this experimental phase.

In addition, before and after (5 days) making electrical characterization, calcium aluminate was characterized by X ray Diffraction (XRD), using a Bragg Brentano geometry for powder diffractometry in a Rigaku Ultima III equipment at a speed of 0.6° 2θ /min in the range 5–42° 2θ, according to reported researches [27,28].

### 3.2. Results

**Figure 4:**
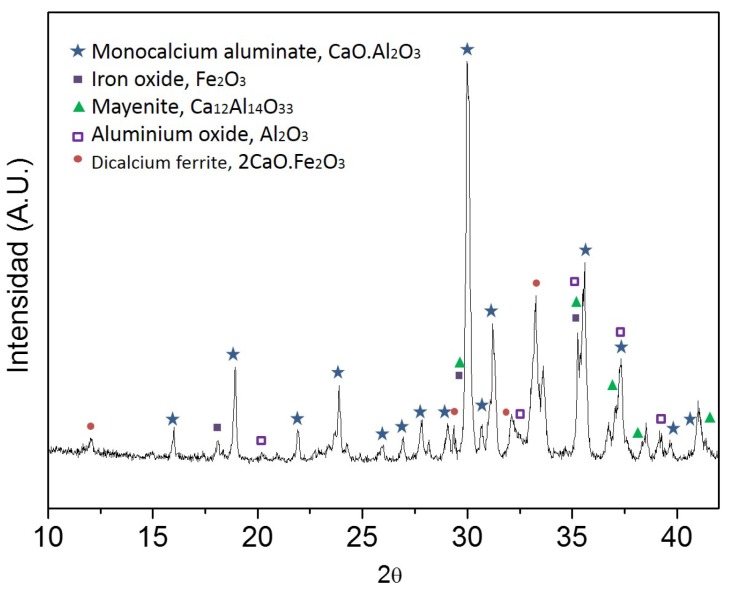
XRD Spectrum of Aluminate Ternal RG.

XRD characterization (Figure 4) demonstrates that the aluminate Ternal RG given by Parexlanko- France presents small traces of iron oxide (Fe_2_O_3_) and other ferrous compounds (i.e., 2CaO·Fe_2_O_3_ that can be the result of high temperatures (greater than 1000 °C) during aluminate production [29]). Also, it shows the presence of monocalcium aluminate, CaO·Al_2_O_3_ (CA) that is the principal compound of the sample, and also little proportion of mayenite, 12CaO·7Al_2_O_3_ (C_12_A_7_), that is indispensable for promoting the setting process of cementitious materials.

**Figure 5:**
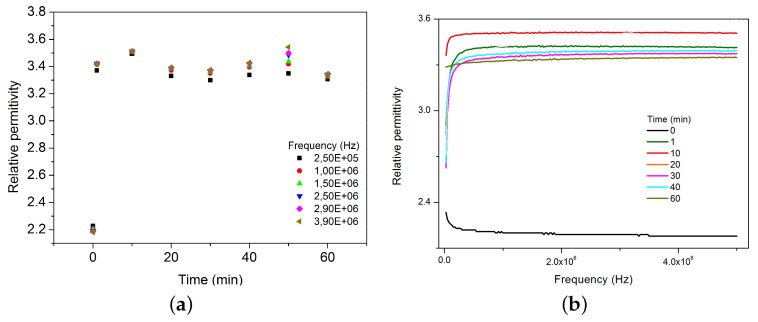
Time effect on CA’s behavior when 0.7 M NaCl solutions are added at specific frequencies. (**a**) Relative permittivity vs Time, regarding specific frequencies and (**b**) Relative permittivity vs. Frequency, regarding time. At 0 min the CA was dried.

In general, all of the tests present the same trend shown in Figure 5. It means, in the first 10 min of each experiment, they reach a steady value. Figure 5 suggests that relative permittivity does not depend on the time nor the frequency at this range, and it is coherent with Figure 2. Considering that interaction depends on diffusion processes, the presence of outliers at time *t* = 50 min could be explained because of that, i.e., preferential flow direction. These results state that polarization mechanisms are constant, and then dielectric behavior is virtually independent of frequency [30,31].

**Figure 6:**
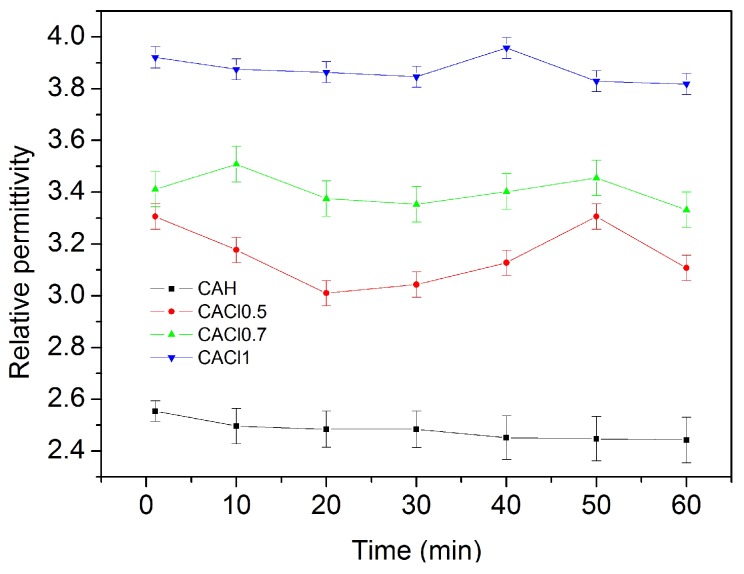
Time effect on CA’s electrical behavior when 0 M, 0.5 M, 0.7 M and 1 M NaCl solutions are added. Each data set is the mean of three.

In contrast, Figure 6 shows the change in relative permittivity as chloride solution concentration increases. The effect of chloride solutions is to increase the relative permittivity. It means that parallel plates capacitance arises due to the ingress of Cl^−^ and Na^+^ ions that caused an ionic polarization inside the material.

**Figure 7:**
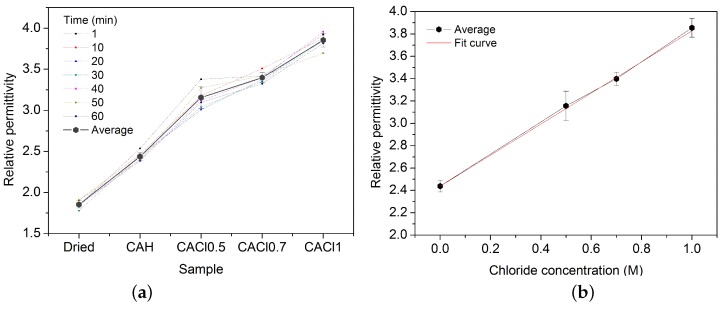
(**a**) Effect of chloride content on measured relative permittivity for CA dried and exposed to 0 M, 0.5 M, 0.7 M and 1 M NaCl solutions; (**b**) Correlation between chloride concentration and relative permittivity.

Figure 7 demonstrates that relative permittivity is linearly dependent on chloride concentration through equation:

(4) $$\varepsilon_{r}=2.438+1.391\chi$$

where: $\varepsilon_{r}$ is the relative permittivity, and χ is the chloride concentration (M).

It means that ionic polarization of NaCl and molecular polarization of H_2_O lead to higher values of the dielectric constant increasing the stored charge in the CA samples.

**Figure 8:**
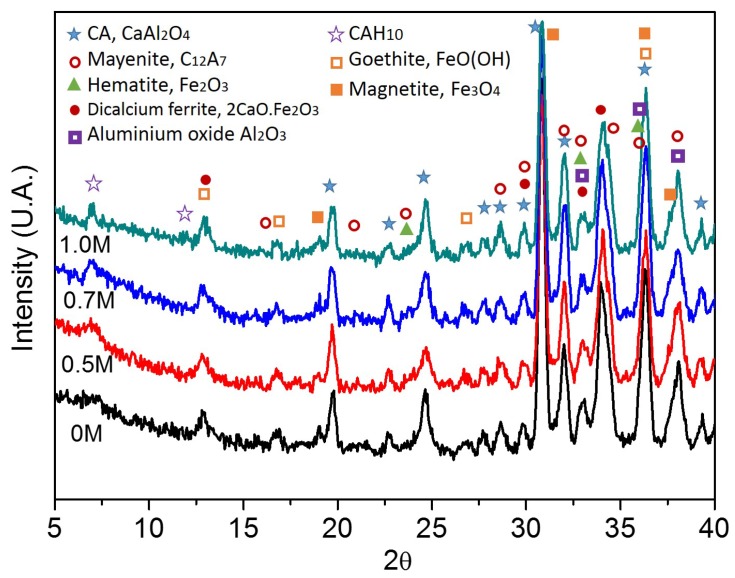
XRD Spectrum of dried aluminate after its interaction with aqueous chloride solutions (0 M, 0.5 M, 0.7 M, 1.0 M).

On the other hand, there is no evidence of Friedel’s salts presence (Figure 8), maybe because the chloride concentration is too low and its proportion with respect to water, CaO and Al_2_O_3_ is not right [32], and because the time for its formation was not enough [33]. However, this result agrees with electrical results, where the most important influence is the ionic polarization. It means that Cl^−^ ions are free inside the material.

Figure 8 states that goethite (13°, 17°, 27°) is present on calcium aluminate surfaces as soon as it gets in contact with water. On the other hand, it is possible to identify the presence of hydrated aluminate (CAH_10_, 6.8° and 11.8°), that is formed by the reaction of water and CA at low temperatures, ∼20 °C [34,35]. Besides, other ferrous compounds are found, i.e., hematite (24°, 33.2°, 35.5° [36]) and magnetite (18.2°, 30°, 35.7°, 37.5° [37]) due to the reaction of oxygen and iron present in the CA.

## 4. Phase II—Chlordetect

**Figure 9:**
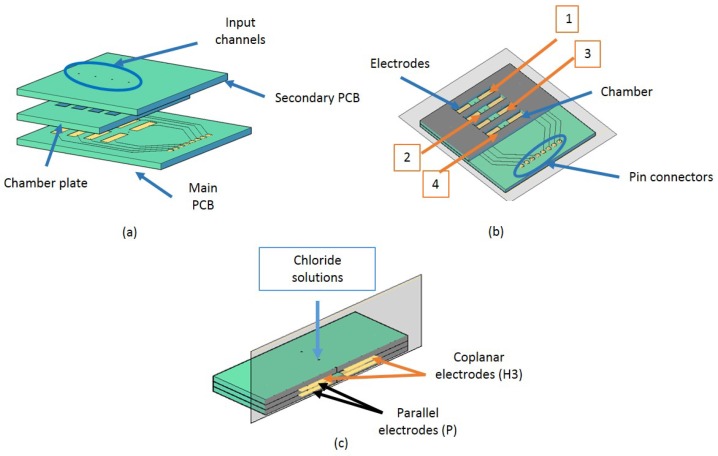
(**a**) sensor design; (**b**) longitudinal section of the device showing the label for each chamber; and (**c**) cross section of the third chamber showing the label of each capacitor according to the chamber.

The proposed device is a conductimetric sensor that consists of two electrodes and a selective layer that interacts/reacts with chloride ions. The device has eight pairs of electrodes (Figure 9). This configuration allows chloride ingress assessment in any structure at different depths, or multiple measurements on the same depth depending on the device position. Additionally, it is possible to measure impedance changes between coplanar electrodes allowing early detection of chloride presence.

The selective layer is monocalcium aluminate (CA) that showed a dependent electrical behavior on chloride presence. It means that electrical properties of CA are proportional to chloride concentration.

### 4.1. Materials

Regarding that the gadget must face different environments exposed to hard conditions (temperature, humidity, corrosive agents), strong materials that withstand the environmental conditions are required. Table 3 lists the materials that are currently used for assembling the device.

FR–4, ID reference used by the National electrical Manufacturers Association (NEMA) for epoxy resin with woven glass as reinforcement, is the most commonly used composite material for electronic technology (e.g., computers, telecommunications, aerospace, industrial controls, automative applications) [38,39].

### 4.2. Performance Tests

The variables that were considered in this phase are summarized in Table 4. The proposed experimental design had two factors: concentration and time, the first one with 20 levels while the second one had 13, and one block with four levels (chambers). According to experimental design theory, this kind of experimental design is called Randomized Blocking Design (RBD), and the statistical model related to this design is:

(5) $$Y_{ijk}=\mu +\tau_{i}+\delta_{j}+{\left(\tau \delta \right)}_{ij}+\beta_{k}+ME_{ijk}\left\{\begin{array}{c} i=1,2,3,\ldots ,20\\ j=1,2,3,\ldots ,13\\ k=1,2,3,4 \end{array}\right.$$

where $Y_{ijk}$ is the response *Z*; μ is a parameter common to all treatments called the overall mean; τ,δ and τδ represent the effects of the factors (i.e., concentration and time) and their interactions; β is the block effect (chamber); and ME is the model error with NID (0, $\sigma^{2}$) (standard normal distribution: average of zero and a constant variance). δ, τ, and β are parameters unique to the *i*th, *j*th or *k*th factors/block and their combinations, and they represent the deviations from the constant when a specific treatment is applied [40].

Using the designed device (Figure 9), now the probe for measuring was the Kelvin Clip 16089A, and the impedance analyzer is still the Agilent 4294A, used in the previous phase. This instrument works in the frequency range of 100 Hz to 100 kHz, at a voltage of 0.5 V, and at 19 ± 1 °C. For this phase the frequency range studied was between 52 kHz and 65 kHz. This reduction on the frequency was possible due to the steady state that commercial CA showed in the previous phase (Figure 6). In addition, this probe did not measure capacitance but impedance, that in this stage is the response under studying.

Regarding the porous and toruous microstructure of the powder, a heterogeneous diffusion inside the chambers can take place. It means, that it is possible that chlorides go inside the chambers with some directionality making parallel capacitors measurements different from those coplanar measurements. Because of this, just the coplanar results are shown in this paper. Figure 9 displays the labelling of each chamber and one example of their names. The others are: H1, H2, and H4 depending on the chamber where the measurement were taken.

By using this experimental design, it is possible to determine sensitivity and response time without forgetting the possible effects of the chamber. Table 5 shows concentrations and times regarded in this study. The selected concentrations correspond to the working range under real conditions, and focusing on small concentrations allows to determine the resolution of the proposed device. All the combinations between them (*Citj*) were performed 4 times (one time per block). In total, 21 devices were used. Additionally, at the beginning of each experiment the impedance of the dried CA (DCA) is measured as a reference ($Z_{0}$).

The results and their analysis are divided into three parts: the first part is focused on the design suitability of the device, the second part explores the overall performance of the device when the CA inside it interacts with chloride solutions, and the third part uses the statistical analysis for determining the sensor’s performance and sensitivity.

### 4.3. Design Suitability

Two different measurements are studied: those made with the device empty (NCA), and those made with the dried CA (DCA) inside the device (dried measurements were done before CA interacts with solutions for all devices). In order to do this, one device (NCA) is analysed, and results from two of the devices are shown as examples of dried CA behavior (D12 and D20); however, statistical analysis is done over all 20 devices (the 21th device was empty).

#### 4.3.1. Empty Device

**Figure 10:**
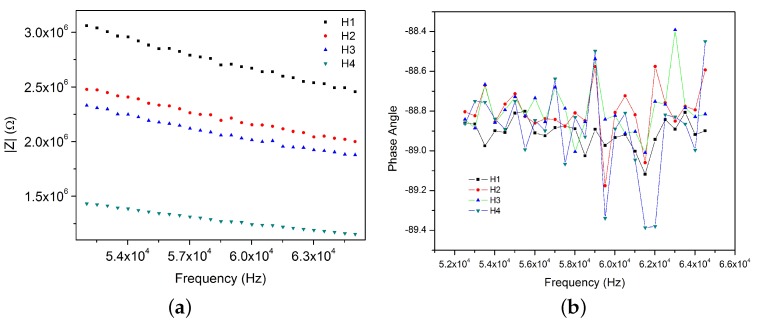
Bode diagrams of coplanar plates capacitors of empty device (NCA) (**a**) Impedance and (**b**) Phase angle.

From coplanar capacitors measurements shown in Figure 10b, to conclude that parallel capacitors are quasi perfect capacitors is possible since in the phase angle diagram the values are near to −90° (approximately 88.8%). It means, that in this frequency range (52 kHz–65 kHz) there is not any electrical resistivity behavior and capacitance values are virtually independent of frequency variation.

In addition, Figure 10a shows a tendency between the measurements. Even when none of them register exactly the same value, their magnitude orders over the frequency do not change (∼10^6^ Ω).

Besides, impedance measurements increase when the chamber is far from the pin connectors; and impedance of chambers two and three (H2 and H3) are closer between them than to the others. This fact can be caused by geometrical or electric factors.

#### 4.3.2. Dried CA

**Figure 11:**
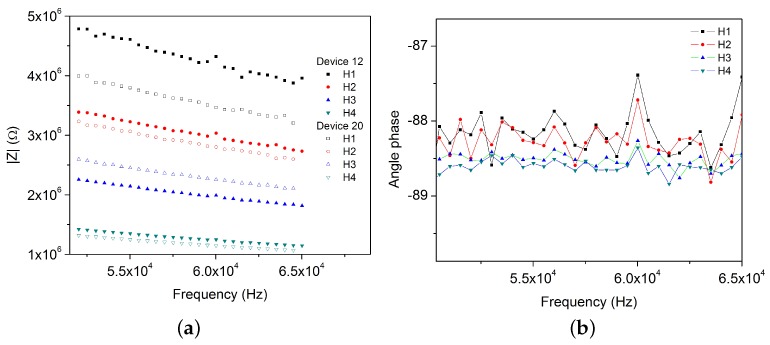
Bode diagrams of coplanar plates capacitors of dried CA (DCA) of devices 12 and 20. (**a**) impedance and (**b**) phase angle.

On the other hand, when devices are filled with CA the impedance increases since the CA is a dielectric material; however, the order of magnitude is the same as that when the device was empty (Figure 11). Besides, capacitors still behave as quasi perfect capacitors (Figure 11b). From both figures it is possible to conclude that measurements are coherent between device 12 and device 20. The measurement behavior is the same than for the empty device: impedance decreases from H1 to H4.

**Figure 12:**
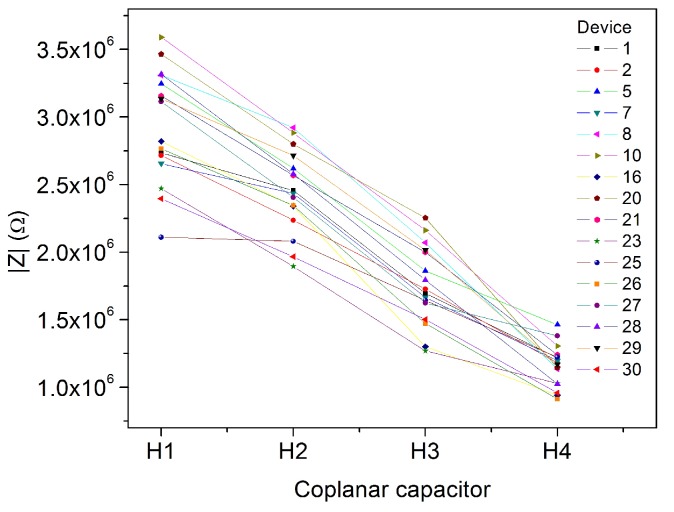
Impedance measured for all the DCA using H1, H2, H3 and H4 at 60,016 Hz.

Regarding the variation of impedance between coplanar electrodes from different chambers, Table 6 shows the mean, standard deviation and coefficient of variation (*COV* = *Std.Dev*/*Mean*) for all 20 measurements made at one frequency (*f* = 60,016 Hz). Figure 12 displays the data for dried CA.

From Table 6 and Figure 12 it is possible to observe that for the same conditions, impedances of H4 are the lowest, this could be due to the electric path that is the shortest one; thus, the impedance due to the path is less than for H1, H2 and H3.

Likewise, the variation between measurements (standard deviation) from the same chamber decreases from H1 to H4. This could be explained since H4 is the closest chamber to the electric pins, it means that the electric path is the smallest one.

Similarly, the COV over the frequency is almost the same for each chamber, and also, the variation is not very different between chambers, since their values are between 12% and 16%.

### 4.4. Overall Performance after Chloride Exposure

#### 4.4.1. Chloride Concentration Effects

Usually, the dielectric constant can be deduced from the signal *Z* vs. Frequency *f* of parallel capacitors (Figure 1) [41,42]. However, as following results show, impedance changes are directly related to chloride presence. It means that a relative impedance (ΔZ) with the dried CA as a reference can give more information about chloride presence than an absolute measurement that is confirmed by the comparison of the response of devices in Figure 10a. ΔZ is calculated by:

(6) $$\Delta Z=Z_{f}-Z_{0}$$

where $Z_{f}$ is the measured impedance and $Z_{0}$ is its respective dried CA impedance.

**Figure 13:**
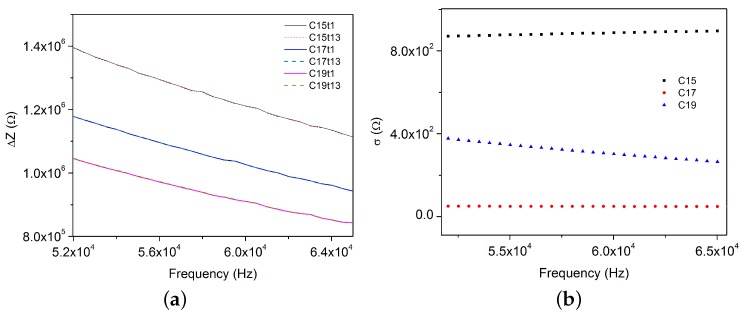
ΔZ vs. Frequency (**a**) Time and (**b**) Standard deviation (σ): C15—0.50%, C17—1.50%, and C19—2.50%.

In general, preliminary results show that impedance difference (ΔZ) between initial dried CA ($Z_{0}$) and CA exposed to solutions (0.50 wt % Cl^−^, 1.50 wt % Cl^−^, and 2.50 wt % Cl^−^) shows a steady state in the chosen frequency range (Figure 13). It is also noted that ΔZ decreases as chloride concentration increases over frequency. This behavior will be analysed in more detail in Section 4.5.

#### 4.4.2. Time Effects

Regarding time response of the same previous treatments, it can be noted that there is not a significant difference between the final impedance at 90 min (t13) and the first impedance at 2 min (t1). This finding is illustrated in Figure 13 (order of magnitude < 10^3^). Figure 13 also shows the standard deviation (σ) of ΔZ over the frequency estimated only taking into account time, as:

(7) $$\sigma =\sqrt{\frac{\Sigma_{j=1}^{13}{\left(Y_{j}-\bar{Y}\right)}^{2}}{13}}$$

where $Y_{j}$ is the measurement in the time *j* and $\bar{Y}$ is the mean of all measurements.

For all measurements shown in Figure 13, the standard deviation could be neglected. This confirms that, like in the characterization phase (Section 3), these measurements are independent over time. This fact changes the statistical model proposed in Section 4.2 Equation (5). The statistical model becomes:

(8) $$Y_{ik}=\mu_{ik}+\tau_{i}+\beta_{k}+ME_{ik}\left\{\begin{array}{c} i=1,2,3,\ldots ,20\\ k=1,2,3,4 \end{array}\right.$$

where $Y_{ik}$ is the response ΔZ, μ is the average of the measurement, τ represents the effect of the concentration factor, β is the block (chamber), and ME is the model error.

On the other hand, Figure 13 also shows that impedance depends on frequency. According to Equation (1), resistance is independent to frequency, and as we see in the previous phase for this frequency range, the capacitance is also independent. Nevertheless, neglecting the frequency variation is impossible because of the factor 1/ω that affects the capacitance and decreases the capacitive reactance value. Nevertheless, frequency can be measured and controlled, and electrical equipments can be used to fix it for future developments.

### 4.5. Performance Tests and Sensitivity

**Figure 14:**
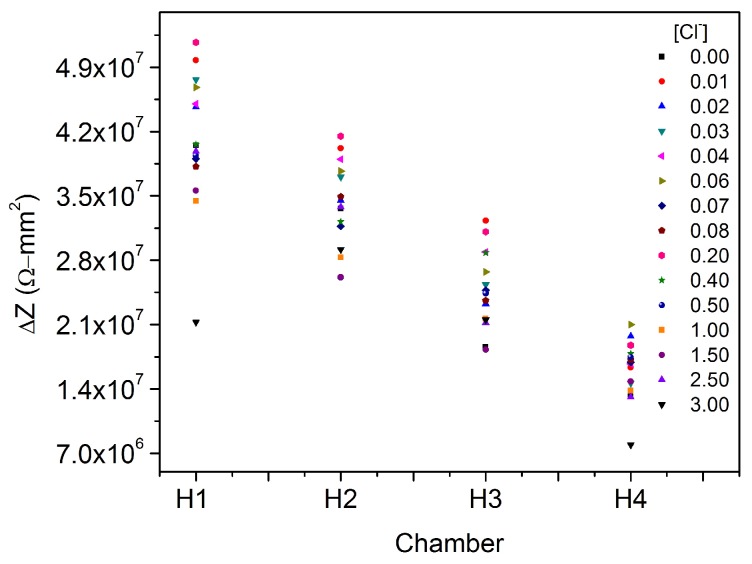
General behavior of ΔZ in presence of chloride solutions according to the chamber where the measurement was taken, the frequency is 60,016 Hz. Concentration units in wt %.

Figure 14 displays the behavior of all coplanar capacitors in presence of 15 different chloride solutions at 60,016 Hz according to the chamber where the measurements were taken (H1, H2, H3 or H4). Note that we have considered just 15 from the original 20 concentrations due to some experimental problems.

The first interesting result is that all the measurements shift according to the chamber from higher to lower impedance differences. This fact is a proof of the effectiveness of the RBD. Likewise, all of the measurements have the same magnitude order (∼10^7^ Ω·mm^2^).

Using the software PASW Statistic 18 (SPSS) the statistical tests of the data shown in Figure 14 were performed for a confidence interval of 95%. Table 7 summarizes the complete analysis of variance of this experiment. This table states that the overall mean μ cannot be neglected(p<0.05). Besides, concentration significantly affects the impedance changes (p<0.05). In addition, it shows that the chamber affects the measurement of the impedance once the solution interacts with the CA (p<0.05). In addition, R squared of 0.928 is in reasonable agreement with the adjusted R squared of 0.899. Their difference is less than 0.20 (0.029) that indicates that there is no problem with the model or/and data.

Likewise, by using the Fisher LSD test for probing $H_{0}$: $\mu_{i}=\mu_{j}$:

(9) $$LSD=t_{\alpha /2,N-a}\sqrt{\frac{2MS_{E}}{n}}$$

where $t_{\alpha /2,N-a}$ is the t-student value for the number of experiments (N=15×4=60), and for the number of treatments (a=15) with a significance coefficient of α=0.05, $MS_{E}$ the mean square error (Table 7), and n the number of blocks (n=4). It is possible to compare the means of the treatments to determine the difference between them and recognizing some parameters of the sensor under lab conditions such as limit of detection and resolution.

According to Table 8 it is possible to say that there is a significant difference (p<0.05) in the system response when 0.01 wt % (*i*th) is added compared when the CA interacts with deionized water (*j*th). Similarly, observing the significance for mean comparison, there is not any difference between 0.01 wt % and 0.02 wt %, 0.03 wt %, 0.04 wt %, and 0.06 wt % (p>0.05). The *p*-value is significant just when the solution is 0.07 wt %, then the difference between the limit of detection (0.01 wt %) and the next concentration that affect the response (0.07 wt %) is 0.06 wt % . This is the resolution of the device.

These results show that the device present a limit of detection of 0.01 wt % Cl^−^, and the resolution, it means, the lowest concentration difference is 0.06 wt % Cl^−^. In addition, they show that the capacity of detection is lower than that observed (0.026 wt % Cl^−^) in the previous phase when CA were characterized, Section 3.

Finally, with the same software it was possible to estimate the parameters for the effects model (Equation (8)) proposed in Section 4.4. Table 9 lists all of the values at 60,016 Hz. According to this table, it is important to highlight that statistically, chamber 4 and 3.00 wt % solution are redundant. It means that their values can be partially or completely deduced from the values of other data items. In addition, the *p*-value for 1.50 wt % Cl^−^ solution is not significant. In addition, taking into account that in the previous Table 8 value 2.00 wt % Cl^−^ shows also a different behavior, it could mean that the upper limit of measuring using the Chlordetect is near to 1.50 wt % Cl^−^. However, for proving this hypothesis it is necessary to perform more experiments under real conditions.

**Figure 15:**
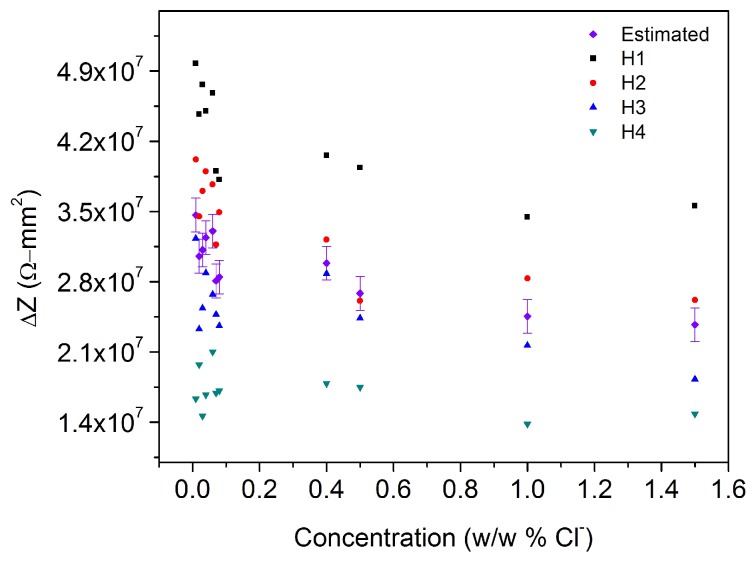
Chloride concentration effect on ΔZ according to the chamber where the measurement was taken, the frequency is *f* = 60,016 Hz.

At the end, it is possible to observe the effect of chloride concentration on impedance difference, disregarding the chambers effects. In other words, the software makes a calculation for estimating the value of the impedance considering just the concentration. Figure 15 displays the behavior for all the chambers at 60,016 Hz with their estimated value and standard error in presence of chloride solutions from 0.00 wt % Cl^−^ to 1.50 wt % Cl^−^. From there it is possible to conclude that impedance difference decreases as chloride concentration increases.

## 5. Conclusions and Challenges for the Future

Experiments show that CA’s dielectric behavior is independent of frequency and time. It means that (i) polarization mechanisms constantly work during and after the contact with chloride solutions, and (ii) interaction between CA and Cl^−^ is fast and reaches a steady state quickly; however, that interaction can be not homogeneous causing shifts in measuring.

It was possible to propose a new application for an existing cementitious material: the commercial CA. The electrical and structural characterization of this material have shown that it can be used as a selective layer in a conductivity sensor whose objective is to sense and to quantify chloride ions in the environment: The Chlordetect.

The design and construction processes of the sensor were proposed, considering not just the CA’s properties, but also those properties of other PCB materials (e.g., copper paths, and FR–4 plates).

In addition, this experimental design allows to determine that chloride concentration significantly affects the relative impedance measured between coplanar electrodes. Likewise, the proposed device has a limit of detection of 0.01 wt % Cl^−^, a sensitivity of 0.06 wt % Cl^−^, and a response time of at least 2 min under lab conditions.

Impedance difference due to chloride presence can be assessed by measuring variations between the electrodes inside the Chlordetect.

For future approaches following factors must be studied:

- Geometry and electrical factors, that affects the measurement according to the chamber where it is taken.
- Selectivity, that represent the specific response of the device to chloride in presence of other ions.
- Robustness, that define the correct functionality of the system in presence of invalid inputs or stressful environmental conditions.

## Figures and Tables

**Table 1:** Tests conditions.

Sample Name	NaCl Concentration	NaCl Concentration
	(M)	(wt %)
CA	0 (Dried)	0
CAH	0 (Hydrated)	0
CACl 0.5	0.5	0.0257
CACl 0.7	0.7	0.0357
CACl 1	1.0	0.0486

**Table 2:** Experimental parameters for CA characterization.

Parameters	Description	Designation	Units
Measured	Continuous	Relative Permittivity ($\varepsilon_{r}$)	
Variable	Quantitative	Concentration (χ)	M
	Quantitative	Time (t)	min
Experimental unit	*m* = 6.6 g	Calcium Aluminate (CA)	
	thickness = 2.055 ± 0.215 mm		

**Table 3:** Current materials.

Component	Material
Matrix	Woven fiberglass reinforcement (FR-4)
Electrodes	Copper
Selective layer	Commercial CA

**Table 4:** Experimental parameters for device suitability tests.

Parameters	Description	Designation	Units
Dependent-Response	Continuous	Impedance (*Z* or Δ*Z*)	Ω or Ω·mm^2^
	Quantitative	Concentration (*Ci*)	wt %
Independent-Factors & Blocks	Quantitative	Time (*tj*)	min
	Categorical	Chamber (*Hk*)	
Experimental unit	*m* = 0.110 ± 0.010 g	Calcium Aluminate (CA)	
	exposed area = 12 mm^2^	inside the device	

**Table 5:** Experimental design for performance tests.

Concetration		Time	
[Cl^−^] (wt %)	Label	t (min)	Label
Empty	NCA	0	t0
Dried CA	DCA	2	t1
0.00	C1	4	t2
0.01	C2	6	t3
0.02	C3	8	t4
0.03	C4	10	t5
0.04	C5	15	t6
0.05	C6	20	t7
0.06	C7	25	t8
0.07	C8	30	t9
0.08	C9	40	t10
0.09	C10	50	t11
0.10	C11	60	t12
0.20	C12	90	t13
0.30	C13		
0.40	C14		
0.50	C15		
1.00	C16		
1.50	C17		
2.00	C18		
2.50	C19		
3.00	C20		

**Table 6:** Analysis of impedance variation of DCA for all the measurements at 60,016 Hz.

Coplanar Electrodes	Mean *	Std. Dev. *	COV
H1	2.94 × 10^6^	4.17 × 10^5^	14.20%
H2	2.45 × 10^6^	3.07 × 10^5^	12.51%
H3	1.75 × 10^6^	2.92 × 10^5^	16.67%
H4	1.16 × 10^6^	1.56 × 10^5^	13.44%

* Units for Mean and Standard Deviation in Ω.

**Table 7:** ANOVA for RBD. Dependent variable: Δ*Z*. *f* = 60,016 Hz.

Source	Sum of Squares	df *	Mean Square	F	*p* **
Intercept	4.92 × 10^16^	1	4.92 × 10^16^	4380.094	<0.001
Concentration	9.00 × 10^14^	14	6.43 × 10^13^	5.72	<0.001
Chamber	5.16 × 10^15^	3	1.72 × 10^15^	153.217	<0.001
Error	4.72 × 10^14^	42	1.12 × 10^13^		
Total	5.57 × 10^16^	60			

* Degree of Freedom, ** Significance. R Squared = 0.928 (Adjusted R Squared = 0.899).

**Table 8:** LSD Test mean comparison: between *i*th concentration and *j*th concentration.

*i*th	*j*th	Mean Diff. (Ω·mm^2^)	Std. Error	*p*
		(*μ*_*i*th_ − *μ*_*j*th_)		
0.010 **	0.00	8.14 × 10^6^ *	2.37 × 10^9^	0.001
	0.02	4.09 × 10^9^	2.37 × 10^9^	0.091
	0.03	3.48 × 10^9^	2.37 × 10^9^	0.15
	0.04	2.25 × 10^9^	2.37 × 10^9^	0.349
	0.06	1.59 × 10^9^	2.37 × 10^9^	0.507
	0.07	6.56 × 10^6^ *	2.37 × 10^9^	0.008
	0.08	6.19 × 10^6^ *	2.37 × 10^9^	0.012
	0.40	4.79 × 10^6^ *	2.37 × 10^9^	0.05
	0.50	7.81 × 10^6^ *	2.37 × 10^9^	0.002
	1.00	1.01 × 10^10^	2.37 × 10^9^	<0.001
	1.50	1.09 × 10^10^	2.37 × 10^9^	<0.001
	2.00	1.94 × 10^9^	2.37 × 10^9^	0.418
	2.50	7.64 × 10^6^ *	2.37 × 10^9^	0.002
	3.00	1.47 × 10^10^	2.37 × 10^9^	<0.001

* The mean difference is significant at the 0.05 level. ** Concentration units in wt % Cl^−^.

**Table 9:** Estimated parameters for effects model for *f* = 60,016 Hz.

Dependent Variable: Δ*Z*				
Parameter	Estimated Parameter	Std. Error	t	*p*
Intercept	7.15 × 10^6^	1.84 × 10^6^	3.893	<0.001
Concentration = 0.00	6.54 × 10^6^	2.37 × 10^6^	2.759	0.009
Concentration = 0.01	1.47 × 10^7^	2.37 × 10^6^	6.193	<0.001
Concentration = 0.02	1.06 × 10^7^	2.37 × 10^6^	4.466	<0.001
Concentration = 0.03	1.12 × 10^7^	2.37 × 10^6^	4.725	<0.001
Concentration = 0.04	1.24 × 10^7^	2.37 × 10^6^	5.245	<0.001
Concentration = 0.06	1.31 × 10^7^	2.37 × 10^6^	5.523	<0.001
Concentration = 0.07	8.12 × 10^6^	2.37 × 10^6^	3.427	0.001
Concentration = 0.08	8.49 × 10^6^	2.37 × 10^6^	3.583	0.001
Concentration = 0.40	9.89 × 10^6^	2.37 × 10^6^	4.174	<0.001
Concentration = 0.50	6.87 × 10^6^	2.37 × 10^6^	2.898	0.006
Concentration = 1.00	4.58 × 10^6^	2.37 × 10^6^	1.933	0.06
Concentration = 1.50	3.74 × 10^6^	2.37 × 10^6^	1.577	0.122
Concentration = 2.00	1.27 × 10^7^	2.37 × 10^6^	5.376	<0.001
Concentration = 2.50	7.03 × 10^6^	2.37 × 10^6^	2.968	0.005
Concentration = 3.00	0 a	-	-	-
Chamber = 1	2.45 × 10^7^	1.22 × 10^6^	20.049	<0.001
Chamber = 2	1.80 × 10^7^	1.22 × 10^6^	14.677	<0.001
Chamber = 3	8.80 × 10^6^	1.22 × 10^6^	7.19	<0.001
Chamber = 4	0 a	-	-	-

t: Statistic value t-student. a: This parameter is set to zero because it is redundant.

## Abbreviations

The following abbreviations are used in this manuscript:

AC	Alternate Current
CA	Monocalcium aluminate
COV	Coefficient of Variation
DC	Direct Current
DS	Dielectric Spectroscopy
ISE	Ion Selective Electrode
PCB	Printed Circuit Board
RBD	Randomized Blocking Design
RC	Reinforced Concrete
XRD	X-ray Diffraction
